# Enhanced candicidal compound production by a new soil isolate *Penicillium verruculosum* MKH7 under submerged fermentation

**DOI:** 10.1186/s12866-016-0713-8

**Published:** 2016-12-09

**Authors:** Shruti Talukdar, Madhumita Talukdar, Manorama Buragohain, Archana Yadav, R. N. S. Yadav, T. C. Bora

**Affiliations:** 1 Biotechnology Division, CSIR-North-East Institute of Science & Technology, Jorhat, 785006 Assam India; 2 Department of Life Sciences, Dibrugarh University, Dibrugarh, 786004 Assam India

## Abstract

**Background:**

Microorganisms are a rich source of structurally diverse secondary metabolites that exert a major impact on the control of infectious diseases and other medical conditions. The biosynthesis of these metabolites can be improved by manipulating the nutritional or environmental factors. This work evaluates the effects of fermentation parameters on the production of a lactone compound effective against *Candida albicans* by *Penicillium verruculosum* MKH7 under submerged fermentation. Design–Expert version8.0 software was used for construction of the experimental design and statistical analysis of the experimental data.

**Results:**

The important factors influencing antibiotic production selected in accordance with the Plackett–Burman design were found to be initial pH, temperature, peptone, MgSO_4_.7H_2_O. Orthogonal central composite design and response surface methodology were adopted to further investigate the mutual interaction between the variables and identify the optimum values that catalyse maximum metabolite production. The determination coefficient (R^2^) of the fitted second order model was 0.9852. The validation experiments using optimized conditions of initial pH 7.4, temperature 27 °C, peptone 9.2 g/l and MgSO_4_.7H_2_O 0.39 g/l resulted in a significant increase (almost 7 fold from 30 to 205.5 mg/l) in the metabolite production which was in agreement with the prediction (211.24 mg/l). Stability of the compound was also assessed on the basis of its response to physical and chemical stresses.

**Conclusions:**

So far as our knowledge goes, till date there are no reports available on the production of antibiotics by *Penicillium verruculosum* through media optimization using RSM. Optimization not only led to a 7 fold increase in metabolite yield but the same was achieved at much lesser time (8–10 days compared to the earlier 12–15 days). The enhanced yield of the antibiotic strongly suggests that the fungus *P. verruculosum* MKH7 can be efficiently used for antibiotic production on a large scale.

## Background

Fungal infections have increased dramatically over the last few decades and this rise in the number of opportunistic fungal infections has stimulated research towards the development of novel antifungal agents [1]. Currently the fourth most common cause of nosocomial infection is the Candida species [2]. Notorious among all the Candida species is the *Candida* albicans [3].

Microbes are one of the most productive sources of natural products from which antibiotics are derived [4]. Fungi, particularly the filamentous ones, are highly potential reservoirs of products with excellent antagonistic activity against human pathogens [5]. A variety of secondary metabolites including antimicrobial substances is reported to have been produced by the fermentation of *Penicillium* species [6–8]. Their production depends upon the nutrition and cultivation conditions of the strains [9]. The nutritional requirements and physical parameters can be managed and controlled to increase the productivity of microbial metabolites [10]. However, these processes are usually quite long and arduous when accomplished using routine techniques such as a one-factor-at-a-time method [11].

In any optimization process, first the screening of the important variables has to be done and subsequently estimation of optimal levels of these factors [12]. Statistical methods are quite advantageous with reference to a rapid identification of the significant factors and also decrease in the total number of experiments [13–16]. Application of statistical methods like Plackett–Burman design (PBD) and response surface methodology (RSM) in process optimization have been reported [17, 18]. The shortcomings of conventional methods have been eliminated by RSM [19–21]. RSM is a fast and effective tool as it reduces the number of experimental trials and also helps to compare the significance of a number of variables at a time.

The objective of the present study was to identify the best conditions for the production of a candicidal compound by *Penicillium verruculosum* MKH7 (active against *Candida albicans*) by statistical approach. Plackett–Burman design (PBD) was used to screen the most significant parameters affecting metabolite production followed by a central composite design (CCD) of response surface methodology (RSM) to identify the optimum levels of the significant factors for maximizing the metabolite production.

## Results

**Fig. 1:**
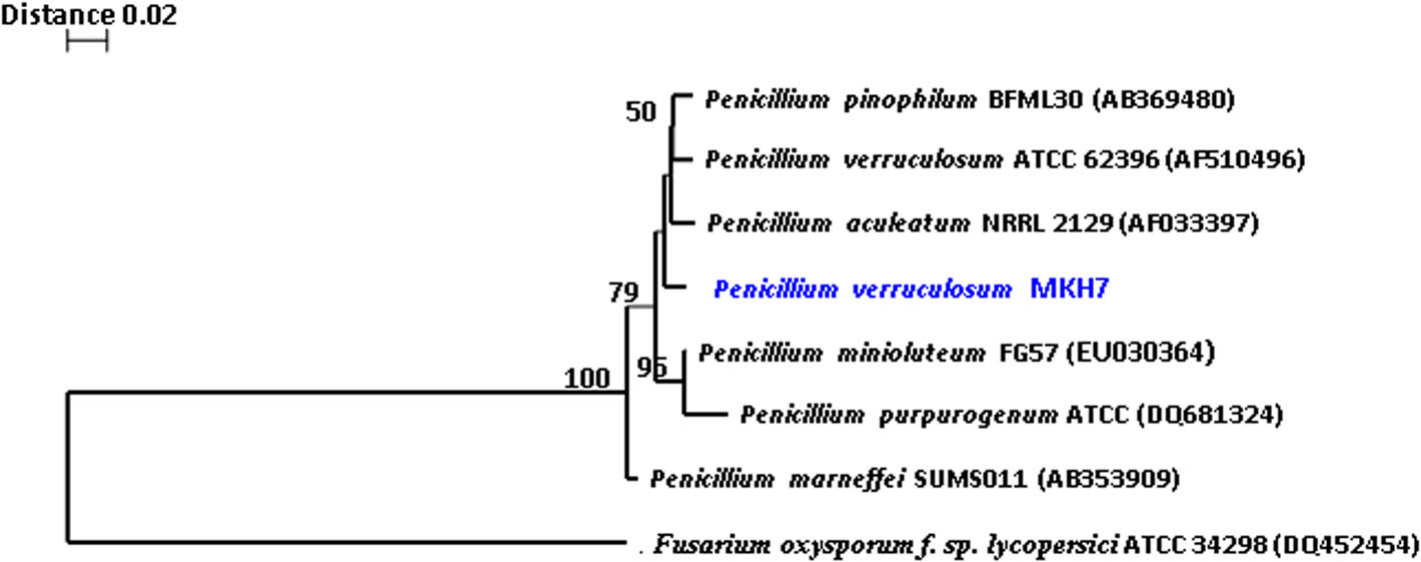
Phylogenetic tree showing relationship Penicillium verruculosum MKH7 and related taxa, based on analysis of ITS region. The numbers on the nodes indicate how often (no. of times, %) the species to the right are grouped together in 100 bootstrap samples. Fusarium oxysporum f. sp. lycopersici ATCC 34298 was used as an outgroup. Bar, 0.05 substitution per site

The promising fungal strain was identified as *Penicillium verruculosum* MKH7 [GenBank: HM049911] on the basis of ITS region sequencing and phylogenetic analysis (Fig. 1). In order to find out the most suitable carbon and nitrogen sources, the one-factor-at-a-time (OFAT) method was employed and on the basis of this, dextrose and peptone respectively were found to be the most favourable for the production of the bioactive metabolite. The composition of the production medium was selected on the basis of preliminary experiments (single factor optimization study, other factors kept constant).

### Characterization of the antifungal metabolite

**Fig. 2:**
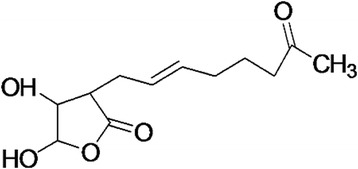
Predicted structure of the antifungal metabolite

The UV spectral data exhibited strong absorption (λ-max) at 200 and 210 nm indicating the presence of lactone ring. The IR spectral absorption at 1721 cm^−1^ and 1751 cm^−1^ revealed the presence of carbonyl group. Other bands that appeared are 3435, 2925, 2850 (OH group –CH_2_-), 1638 cm^−1^(non-conjugated double bond). In the NMR spectrum the signals are δ1.76 (s), δ2.64, δ3.90, δ4.81, δ5.05, δ5.13, δ5.20 and δ5.48. The ^13^C NMR peaks at 176.19, 176.51 and 165.24 strongly favors the presence of carbonyl groups. Other prominent peaks are 64.69, 94.15 and 94.33 indicating the presence of a lactone ring. The alkene carbons are also found to be present at 110.93, 121.61 and 144.90. The remaining methyl and methylene carbons are found to be present at 21.03 (CH_3_), 22.40, 23.14, 38.36, 45.25 (CH_2_ peaks). The C, H, N analysis gave the estimation of carbon (56.45%), hydrogen (7.91%) and nitrogen was negligible (0.78%). Based on the above data, the structure was deduced as 4, 5- dihydroxy-3- (7-oxo-oct-2-enyl)-dihydro-furan-2-one (Fig. 2).

### Optimization by Plackett-Burman design

The significance of the seven factors *viz.* dextrose, peptone, NaCl, initial pH, temperature, fermentation time, MgSO_4_.7H_2_O for metabolite production was examined by Plackett-Burman design. The levels of the factors in the design are given in Table 1. Table 2 shows the experimental design with the seven factors under investigation as well as the effect of each factor on the response and significant levels. Twelve runs were carried out to screen the effect of the variables (including 5 dummy variables) on metabolite production. A first-order model was fitted to the results obtained from the 12 experiments:

**Table 1:** Levels of the factors tested in the Plackett-Burman design

Factor	Code level		
	−1	0	+1
A-Dextrose (g/l)	20	25	30
B-Peptone (g/l)	5	7.5	10
C-Initial pH	5	7	9
D-Temperature (°C)	25	30	35
E-NaCl (g/l)	1	3	5
F-Fermentation time (days)	10	12.5	15
G- MgSO_4_.7H_2_O (g/l)	0.1	0.3	0.5

**Table 2:** Plackett-Burman design matrix for evaluating significant factors affecting candicidal metabolite production by *Penicillium verruculosum*

Run	A	B	C	D	E	F	G	H	I	J	K	Yield (mg/l)
1	1	1	-1	1	1	1	-1	-1	-1	1	-1	3.1
2	1	-1	-1	-1	1	-1	1	1	-1	1	1	3.3
3	-1	-1	-1	1	-1	1	1	-1	1	1	1	3.2
4	1	-1	1	1	1	-1	-1	-1	1	-1	1	3.7
5	-1	1	1	-1	1	1	1	-1	-1	-1	1	30.5
6	-1	1	1	1	-1	-1	-1	1	-1	1	1	3.5
7	1	-1	1	1	-1	1	1	1	-1	-1	-1	6.4
8	-1	1	-1	1	1	-1	1	1	1	-1	-1	15.5
9	1	1	1	-1	-1	-1	1	-1	1	1	-1	18.0
10	-1	-1	1	-1	1	1	-1	1	1	1	-1	15.2
11	-1	-1	-1	-1	-1	-1	-1	-1	-1	-1	-1	3.6
12	1	1	-1	-1	-1	1	-1	1	1	-1	1	3.5

*H, I, J, K- Dummy variables

1 $$ \mathrm{Y}\ \left(\mathrm{mg}/\mathrm{l}\right) = 9.13\ \hbox{-}\ 2.79\ \mathrm{A} + 3.23\ \mathrm{B} + 3.76\ \mathrm{C}\ \hbox{-}\ 3.23\ \mathrm{D} + 2.76\ \mathrm{E} + 1.19\ \mathrm{F} + 3.69\ \mathrm{G} $$

The *t*-test and *P*-values were used to identify the effect of each factor on metabolite production. A P-value of less than 0.05 indicates that the model terms are significant. Table 3 shows that peptone, initial pH, temperature and MgSO_4_.7H_2_O are the most significant factors (*P* < 0.05). These were then selected for further optimization to obtain a maximum response. The fitness of the model was determined by the coefficient of determination *R* ^2^, which in this case was 0.9238.

**Table 3:** ANOVA of the Plackett-Burman Design

Source	SS	DF	MS	*F*-value	*P*-value Prob > *F*
Model	784.52	7	112.07	6.93	0.0401
Dextrose	93.52	1	93.52	5.78	0.0740
Peptone	124.81	1	124.81	7.72	0.0499
Initial pH	169.50	1	169.50	10.48	0.0317
Temperature	124.81	1	124.81	7.72	0.0499
NaCl	91.30	1	91.30	5.65	0.0763
Fermentation time	17.04	1	17.04	1.05	0.3627
MgSO_4_.7H_2_O	163.54	1	163.54	10.11	0.0335

*SS sum of squares, *DF* degrees of freedom, *MS* mean square

### Response surface analysis

Based on the Plackett–Burman design, response surface methodology (RSM) using CCD was employed to determine the optimal levels of the four most significant factors (initial pH, temperature, peptone and MgSO_4_.7H_2_O) for enhancing metabolite production. The four independent variables were studied at five different levels (−2, −1, 0, +1, +2) and a set of 30 experiments with different combination of the selected variables were carried out. The lowest and the highest values of the variables were: initial pH, 7 and 8; temperature, 25 and 30 °C; peptone, 7.5 and 10 g/l; MgSO_4_.7H_2_O, 0.3 and 0.5 g/l. The actual yield of the bioactive metabolite and the yield predicted by the model equation are given in Table 4. Regression analysis was performed to fit the response function (metabolite production) with the experimental data. The second-order polynomial equation for metabolite production is

**Table 4:** Central Composite Design in coded units

Run	Initial pH (A)	Temperature (B)	Peptone (C)	MgSO_4_.7H_2_O (D)	Metabolite yield (mg/l)
1	0	0	0	0	217.56
2	−1	1	−1	−1	70.34
3	0	0	0	2	67.8
4	−1	1	−1	1	73.78
5	1	−1	1	−1	73.78
6	1	1	−1	1	55
7	0	0	0	0	210.7
8	1	1	1	1	32.15
9	0	−2	0	0	71.32
10	0	0	0	0	210.65
11	0	0	0	0	217.5
12	1	1	−1	−1	66.2
13	−1	−1	1	−1	77.7
14	0	0	2	0	80.25
15	1	−1	1	1	75.2
16	0	0	0	0	205.5
17	1	1	1	−1	75
18	1	−1	−1	1	80.14
19	−1	1	1	1	62
20	2	0	0	0	80
21	−1	−1	1	1	66.5
22	−1	−1	−1	−1	78.5
23	0	0	0	0	205.46
24	0	0	0	−2	77.58
25	0	0	−2	0	51.3
26	−1	−1	−1	1	70.85
27	1	−1	−1	−1	46.76
28	0	2	0	0	48
29	−2	0	0	0	70
30	−1	1	1	−1	78.45

2 $$ \mathrm{Y}\left(\mathrm{mg}/\mathrm{l}\right)=211.23\hbox{-} 2.25\;\mathrm{A}\hbox{-} 4.3\;\mathrm{B}+2.4\;\mathrm{C}\hbox{-} 2.9\;\mathrm{D}\hbox{-} 2.41\mathrm{AB}+1.05\ \mathrm{A}\mathrm{C}+0.79\ \mathrm{A}\mathrm{D}\hbox{-} 2.17\;\mathrm{B}\mathrm{C}\hbox{-} 5.2\;\mathrm{B}\mathrm{D}\hbox{-} 5.44\;\mathrm{C}\mathrm{D}\hbox{-} 34.2{\mathrm{A}}^2\hbox{-} 37.99{\mathrm{B}}^2\hbox{-} 36.5{\mathrm{C}}^2\hbox{-} 34.74{\mathrm{D}}^2 $$

where A, B, C and D are the coded factors of initial pH and temperature, peptone and MgSO_4_.7H_2_O respectively.

**Fig. 3:**
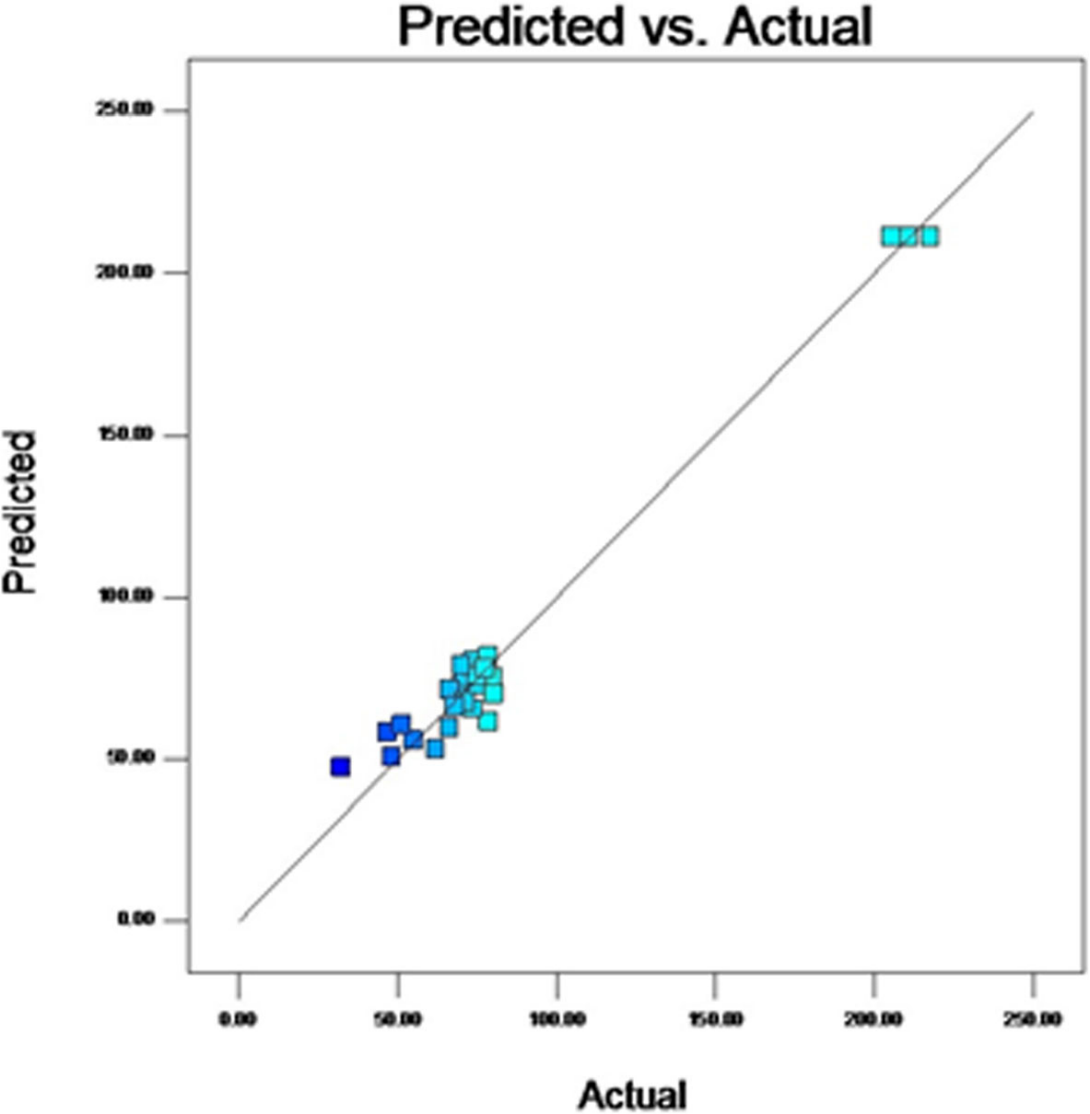
Plot of the observed versus the predicted response

**Fig. 4:**
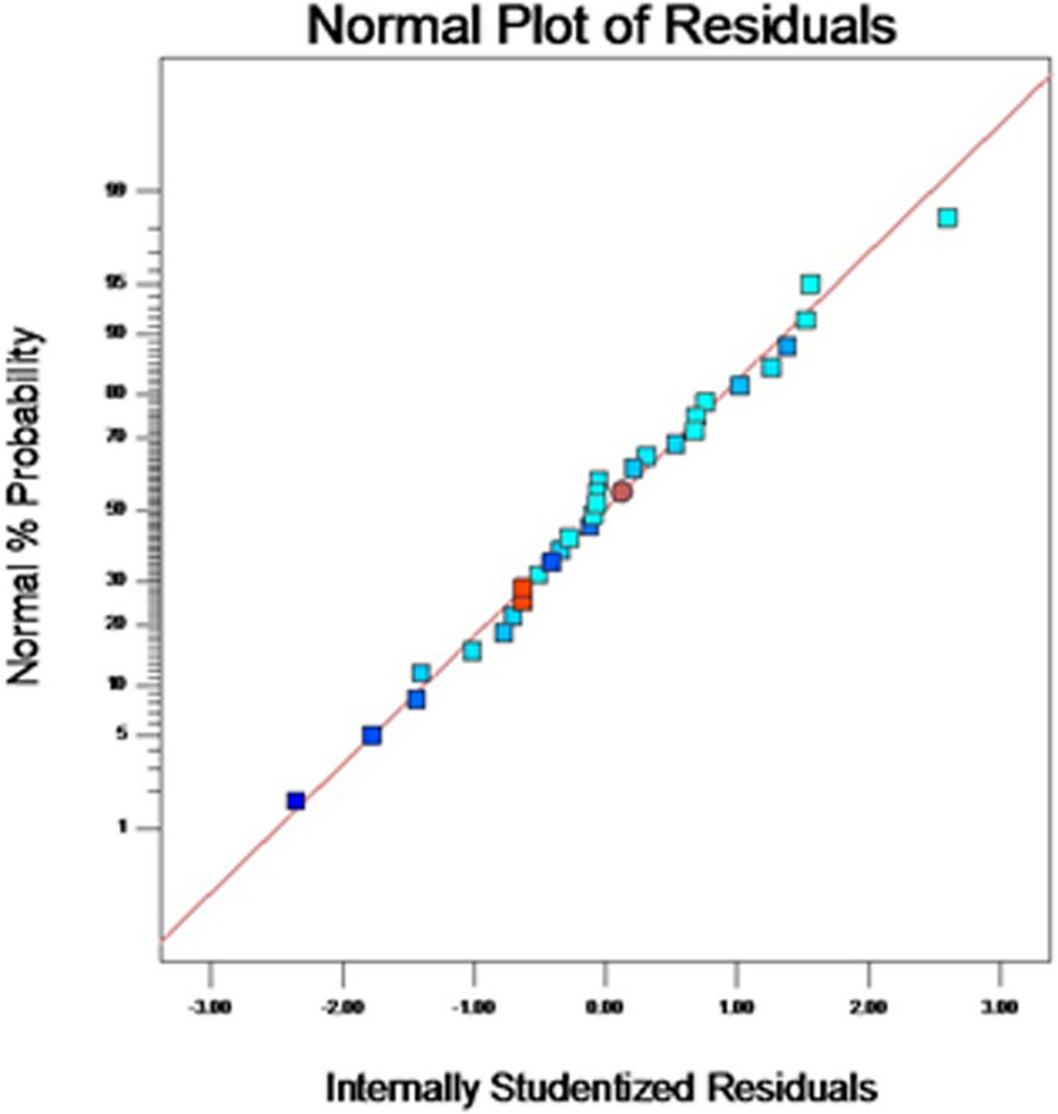
Normal probability of internally studentized residuals

**Fig. 5:**
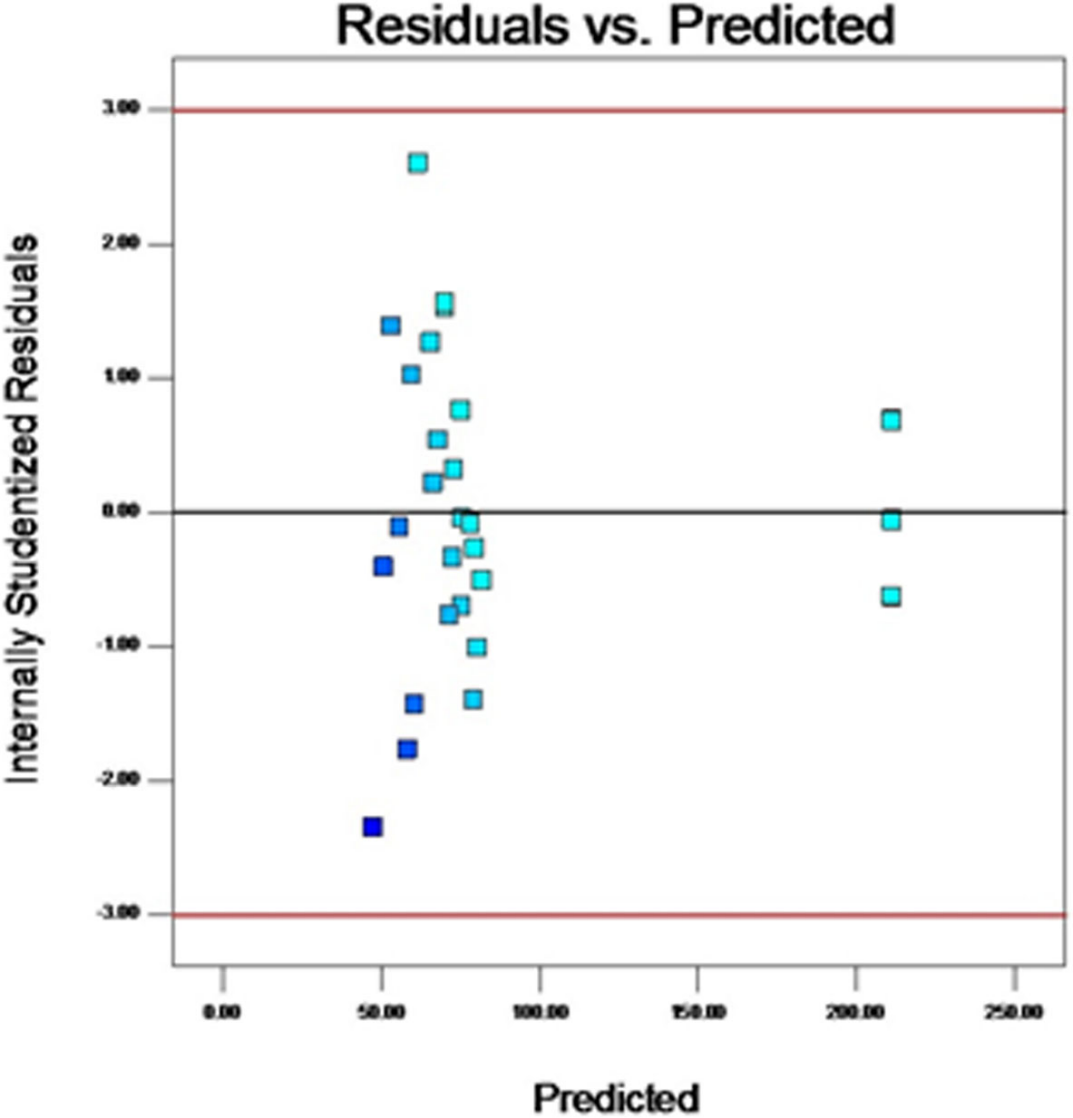
Plot of internally studentized residuals vs. predicted response

**Fig. 6:**
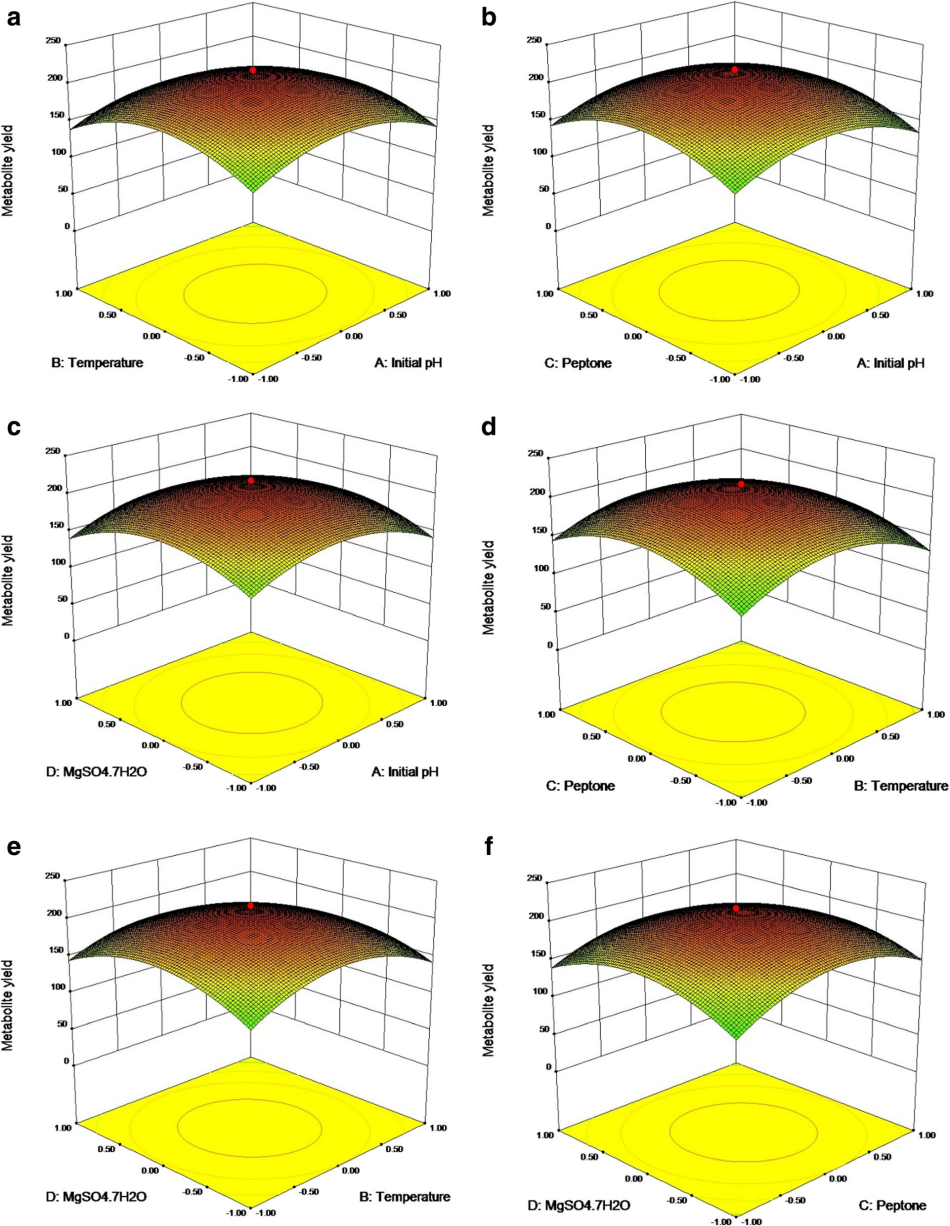
Response surface curve showing the various interaction effects. **a** Initial pH and temperature (**b**) Initial pH and peptone (**c)** Initial pH and MgSO_4_. 7H_2_O (**d)** Temperature and peptone (**e**) Temperature and MgSO_4_. 7H_2_O (**f**) Peptone and MgSO_4_. 7H_2_O

From the results of *F*-test analysis of variance (Table 5), it was found that the regression was statistically significant (*P* < 0.0001). The Model *F*-value of 71.36 implies the model is significant. Values of “Prob > F” less than 0.05 indicate model terms are significant. In this case CD, A^2^, B^2^, C^2^, D^2^ are significant model terms. The fit of the model was checked by the coefficient of determination R^2^ which was calculated to be 0.9852**.** The R^2^-value is always between 0 and 1, and a value >0.75 indicates aptness of the model. The “Pred R-Squared” of 0.9210 is in reasonable agreement with the “Adj R-Squared” of 0.9714. The “Lack of Fit *F*-value” of 4.69 implies the Lack of Fit is not significant. As evident from the linear coefficient of 2.4, the effect of peptone was the highest on the yield of the metabolite while low *P* values (*P* < 0.05) indicated that the interaction between peptone and MgSO4.7H2O was significant [22]. Statistical analysis helps to ascertain the factors generating signals that are large in comparison to noise [23]. Adequate precision measures the signal to noise ratio and a ratio greater than 4 is desirable. In this case, the ratio of 23.049 for antibiotic yield indicates an adequate signal. From the maximum point of the model, the optimal values of the four significant components were calculated to be A = −0.032, B = −0.054, C = 0.04 and D = −0.0423 (in coded units) which corresponds to the values of 7.4, 27 °C, 9.2 g/l and 0.39 g/l for initial pH, temperature, peptone, MgSO_4_.7H_2_O respectively. The model predicted a maximum response of 211.24 mg/l metabolite yield at this point which was significantly higher than that obtained using the original medium. The observed versus the predicted response is shown in Fig. 3. The predicted data from the model are in agreement with the observed one as is evident from the figure. Figure 4 shows the normal plot of residuals, the linear pattern indicating normality in the error term [24]. A plot of residuals versus the predicted response is shown in Fig. 5. As the residuals are scattered randomly so the variance of the original observation is constant for all the values of Y [24]. The interaction between two variables by keeping the other two at zero level are depicted in the 3D response surface curves (Fig. 6).

**Table 5:** Regression results of the CCD

Factor	Coefficient estimate	*F*-value	Prob > *F*	Significance
Intercept	211.23			
Model		71.36	<0.0001	Significant
A	−2.25	1.20	0.2911	
B	−4.3	4.39	0.0536	
C	2.4	1.34	0.2643	
D	2.9	2.06	0.1718	
AB	−2.41	0.92	0.3529	
AC	1.05	0.18	0.6811	
AD	0.79	0.098	0.7581	
BC	−2.17	0.74	0.4024	
BD	−5.2	4.26	0.0567	
CD	−5.44	4.69	0.0469	
A^2^	−34.2	316.76	<0.0001	
B^2^	−37.99	391.87	<0.0001	
C^2^	−36.5	360.97	<0.0001	
D^2^	−34.74	327.56	<0.0001	

*Lack of Fit = 4.69, Coefficient of Variation (%) = 10.41, R^2^ = 0.9852, Adj R-Squared = 0.9714, Pred R-Squared = 0.9210

### Validation of the optimized condition

Validation of the statistical model and regression equation was performed by running the optimization program with Design Expert within the experiment range investigated. Validation experiment was performed in triplicate tests.

### Stability of the metabolite

**Fig. 7:**
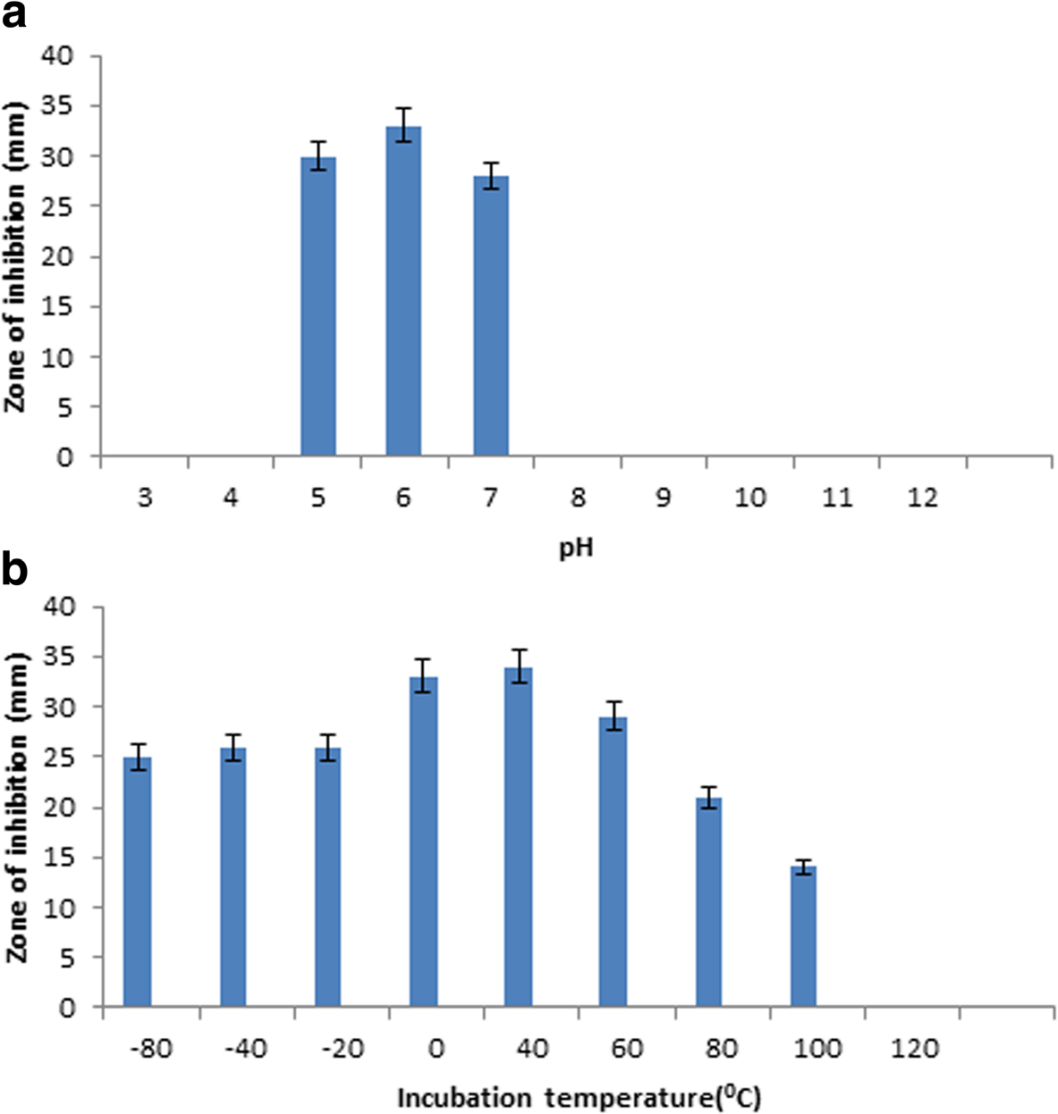
Antibiotic stability in different pH (**a**) and temperatures (**b**)

The response of the bioactive metabolite to physical and chemical stresses was examined. It was stable at low temperatures ranging from −80 to 60 °C for 6 h. There was a moderate decrease in activity on heating the metabolite to 100 °C. There was complete loss of activity upon exposure to temperatures beyond 100 °C. Compound was susceptible to extreme values of pH, being most stable in the range of 5–7. Results are presented in Fig. 7.

## Discussion

Genus *Penicillium* is a potential producer of a vast array of secondary metabolites like tremorgenic toxins, pigments, potentiators of nerve growth factor, inducers of osteoblast differentiation and most importantly antibiotics including macrocyclic polylactones [25–30]. In this report, characterization of the candicidal compound revealed the presence of lactone ring and carbonyl groups as prominent components. Breinholt et al. [27] had also reported the isolation of antifungal macrocyclic polylactones from *P. verruculosum*. Production of secondary metabolites is influenced by a number of cultivation parameters ranging from precursors to trace elements [31]. The combined effects of all the involved factors cannot be determined through traditional practices like the classical one-factor-at-a-time approach [32]. Interactions among factors and multifactor optimization cannot be evaluated using this method. These limitations can be overcome by statistical tools like RSM that enable the study of the effects of several factors simultaneously and determine the optimum values of the variables so as to maximize the response [33, 34]. PBD was applied to determine the main factors affecting metabolite production. In this investigation, from the PBD analysis it was found that among the variables tested, four factors *viz*. initial pH, temperature, peptone and MgSO_4_.7H_2_O, were found to have significant effect on the production of bioactive metabolite by the strain *P.verruculosum* MKH7. Medium pH, temperature [35] and peptone [36] have been reported to be some of the most important experimental parameters influencing bioactive secondary metabolite production by *Penicillium* species. Dextrose and peptone were found to be important also in the production of sclerotiorin from *P.* sclerotiorum [8]. However Brian et al. [30] reported that glucose was the best carbon source for wortmannin synthesis from *P. wortmanni* while glycerol was most favourable for mevastatin production by *P.* citrinum [37]. The reason for this dissimilarity might be the use of different strains and different culturing conditions, yet another reason might be the selection of different carbon sources in the original medium [24]. Moreover, the sources for growth and secondary metabolism may be different. For example, glucose may be beneficial for growth but the same may not be true for secondary metabolite formation [38]. Peptone played a crucial role in the biosynthesis of the metabolite sclerotiorin by *P. sclerotiorum* [39]. Several factors influence the effect of nitrogen sources on synthesis of secondary metabolite. The sources that are important for growth may negatively affect secondary metabolic pathways; there are reports on the negative effects of ammonium salts [38].

Under the RSM optimized conditions of initial pH7.4, temperature 27 °C, peptone 9.2 g/l and MgSO_4_.7H_2_O 0.39 g/l, a maximum metabolite production of 211.24 mg/l was predicted, simultaneously proved by triplicate experiments conducted under the same conditions. MgSO_4_.7H_2_O was found to be essential also for the synthesis of mevastatin from *P.* citrinum [37]. Trace elements like Zn, Mn, Fe, Cu are important for microbial growth because of their presence in metalloenzymes or as enzyme activators [24] while Mg and Ca are macronutrients in fungal nutrition [37]. Contrary to this, in our case the effect of Mg on metabolite yield was significant. Similar inference was also made on the production of sclerotiorin by *P.* sclerotiorum [8]. Temperature and pH affect the regulation of molecules like ATP which in turn influence the regulation of metabolic pathways, coupled reactions and functional yields at the membrane and cell wall level [39]. A change in the concentration of hydrogen ions may change the redox fluxes and oxidative state of energy molecules like ATP, thereby causing diverse metabolism and generating different products [40]. Brian, 1946 [41] reported that high initial pH (>6) of media was best for development of fungistatic activity by *Penicillium terlikowskii*. Similar observation was also made for the production of antimicrobial agent by *P.* viridicatum [42]. Metabolic activity of fungus may be terminated by low temperature while high temperature kills the fungal cell [43]. The optimal temperature range for the production of the antibiotic and nephrotoxin, citrinin, by *P. viridicatum* was 25–30 °C [44]. Previous studies have shown that a temperature of 25 °C was found to be optimal in a number of cases [43].

The use of polyene antibiotics in clinical practice is restricted due to problems in their stability which is affected by extreme values of pH and temperature leading to total loss of drug potency. The stability of lactone antibiotics depends on the tetraene chromophore of the molecule and heating beyond 100 °C leads to cleavage of these four conjugated double bonds resulting in the degradation of the antibiotic [45]. According to Stark, 2000 [46], neutral aqueous suspensions of natamycin can remain stable at 50 °C for several days and a slight decrease in biological activity was observed after heating for 20 min at 110 °C. The polyene antifungal agent nystatin is more active at low temperature (30–25 °C) while amphotericinB is at 41 °C and their activity against *C. albicans* is stable at pH between 5 and 7 [47]. Similar observation was made by Raab, 1972 [45], on the stability of natamycin at different pH. Likewise, phoslactomycin B is most stable at a pH of 6.63 [48]. High pH results in saponification of the lactone and additional decomposition due to a series of retroaldol reactions while low pH might bring about the hydrolysis of the glycosidic bond [49].

So far as our knowledge goes, till date there are no reports available on the production of antibiotics by *Penicillium verruculosum* through media optimization using RSM. The enhanced yield of the antibiotic strongly suggests that the fungus *P. verruculosum* MKH7 can be efficiently used for antibiotic production on a large scale. Optimization not only led to a 7 fold increase in metabolite yield but the same was achieved at much lesser time (8–10 days compared to the earlier 12–15 days). In conclusion, statistical methods can be effectively utilized for arriving at optimal solutions and in analysing the interactive effects of the parameters thereby leading to improved metabolite production.

## Conclusion

This is the first report on the production of antifungal compound by *Penicillium verruculosum* through media optimization using RSM. From the Plackett–Burman design analysis it was found that among the variables tested, four factors *viz*. initial pH, temperature, peptone and MgSO_4_.7H_2_O, were found to have significant effect on the production of bioactive metabolite by the strain *P.verruculosum* MKH7. The RSM optimized conditions of initial pH7.4, temperature 27 °C, peptone 9.2 g/l and MgSO_4_.7H_2_O 0.39 g/l predicted a maximum metabolite production of 211.24 mg/l which was proved by triplicate experiments conducted under the same conditions. A 7 fold increase in metabolite yield was obtained at a much lesser time. In conclusion, statistical methods can be effectively utilized for arriving at optimal solutions and in analysing the interactive effects of the parameters thereby leading to improved metabolite production.

## Methods

### Producer organism

Assay of antibiotic study by agar well diffusion method [50] was carried out to select the strain having the most promising candicidal activity. The test organism used was *Candida albicans* (MTCC 3017) obtained from Microbial Type Culture Collection (MTCC) and Gene Bank, Institute of Microbial Technology (IMTECH), Chandigarh, India. Based on this study, the strain MKH7 isolated from soil collected from North-East India, one of the Megabiodiversity Hot spots, was selected for a detailed analysis to optimise the culture parameters. Disc diffusion method [51] was followed to confirm the efficacy of the compound isolated from MKH7.

### Identification of the strain

The strain was identified by ITS region sequencing. Mycelia was ground under liquid nitrogen and DNA extraction was done from 4-day-old cultures using a HiPurATM plant genomic DNA Miniprep purification spin kit (HimediaR). Amplification of the ITS region of the fungal isolate was performed by forward primer ITS1 5-TCC GTA GGT GAA CCT GCG G-3and reverse primer ITS4 5-TCC TCC GCT TAT TGA TAT GC-3|. The PCR reaction was carried out in a 50-μL reaction mixture containing 50 ng genomic DNA, 10 pmol of each primer, 0.5 mM of dNTPs, 1× PCR buffer with 1.5 mM MgCl2, and 3 U Taq polymerase. The conditions consisted of an initial denaturation at 94 °C for 5 Min, followed by 35 amplification cycles at 94 °C for 1 Min, 54 °C for 1 Min, and 72 °C for 2 Min, and a final extension at 72 °C for 8 Min. The PCR product was electrophoresed on 1.2% agarose gel and desired band of 600 bp excised. DNA from the gel slice was eluted using a GeneJETTM Gel Extraction kit (Fermentas, India) according to the manufacturer’s instructions. The sequence of the PCR product was determined by employing the ABI Prism Big Dye Terminator v. 3.1 Cycle Sequencing kit. The sequence elucidation was performed on a 3730× genetic analyzer (Applied BiosystemsTM, Foster City, CA, USA). Analysis of the sequences was done using the gapped BLASTn (http://www.ncbi.nlm.nih.gov) search algorithm. The evolutionary distances among the fungal strains and their related taxa were calculated with Molecular Evolutionary Genetics Analysis (MEGA) software version 4.0. (CopyrightC Sudhir Kumar, Centre for Evolutionary Functional Genomics, 2007) using Kimura’s two-parameter model, after aligning the sequences with ClustalW. The ITS region sequence of the potent strain MKH7 was submitted to the Gene Bank and assigned the accession number HM049911.

### Chemicals

All media components and chemicals used were of highest purity grade available commercially from Sigma (St. Louis, USA) and Hi-media Ltd., India.

### Media and cultivation

The stock culture of the filamentous fungus was maintained on potato dextrose agar slants. The slants were inoculated with mycelia and incubated at 25 °C for 7 days, and then stored at 4 °C. Seed culture was grown in 250 ml flasks containing 50 ml of basal medium (20 g/l dextrose, 4 g/l peptone, 0.5 g/l K_2_HPO_4_, 0.5 g/l KH_2_PO_4_, 0.5 g/l MgSO_4_. 7H_2_O) at 25 °C for 4 days on a rotary shaker incubator (Kuhner, Switzerland) at 200 rpm. The subsequent flask culture experiments were performed in 250 ml flasks containing 50 ml of the media after incubating with 5% (v/v) of the seed culture under the conditions described above.

### Isolation of metabolites

Metabolite isolation and purification was done by the method described by Breinholt et al. [27]. The solvent layer was evaporated in a rotary evaporator (Buchi 114, Germany) under high vacuum. Bioassay-guided fractionation was followed to pool out the fraction having antagonistic activity against *C. albicans*. The active compound was identified following analytical tools such as IR, NMR, GCMS, and UV. A Perkin Elmer System 2000 FTIR spectrometer was used to record the IR spectra. The UV absorption spectrum was measured with an Analytik Jena UV–Vis Specord200 spectrophotometer and the operating software was Aspect plus v1.7. 1H NMR (300 MHz) and 13C NMR (75 MHz) spectra were obtained with a Bruker AVANCE DPX 300 NMR spectrometer in CDCl3 using TMS as the internal standard. Mass spectra were recorded on a Bruker Esquire 3000 system.

### Experimental design and statistical analysis

#### Plackett-Burman factorial design

Plackett-Burman designs are very efficient screening designs when only main effects are of interest. The independent variables of the culture conditions were dextrose, peptone, NaCl, intial pH, temperature, fermentation time, MgSO_4_.7H_2_O. To determine the key fermentation parameters significantly affecting metabolite production, Plackett-Burman design was employed. Based on the Plackett-Burman factorial design, each factor was tested at low (−1) and high (+1) levels. Plackett-Burman experimental design is based on the first-order polynomial model:

3 $$ \mathrm{Y}={\upbeta}_0+\sum {\upbeta}_{\mathrm{i}}{\mathrm{X}}_{\mathrm{i}} $$

where Y is the response (productivity), β_0_ is the model intercept, β_i_ is the linear coefficient, and X_i_ is the level of the independent variable.

#### Central composite design (CCD) and response surface analysis

To enhance the production of the active metabolite, the 4 most significant factors *viz.* initial pH, temperature, peptone and MgSO_4_.7H_2_O (screened by Plackett-Burman design) were optimized by employing CCD of response surface methodology. The CCD experimental results were fitted with a second-order polynomial equation and a multiple regression of the data was carried out for obtaining an empirical model related to the most significant factors. The general form of the second-order polynomial equation is

4 $$ \mathrm{Y}={\upbeta}_0+\sum {\upbeta}_{\mathrm{i}}{\mathrm{X}}_{\mathrm{i}}+\sum {\upbeta}_{\mathrm{i}\mathrm{i}}{{\mathrm{X}}_{\mathrm{i}}}^2+\sum {\upbeta}_{\mathrm{i}\mathrm{j}}{\mathrm{X}}_{\mathrm{i}}{\mathrm{X}}_{\mathrm{j}} $$

where Y is the predicted response, X_i_ and X_j_ are independent factors, β_0_ is the intercept, β_i_ is the linear coefficient, β_ii_ is the squared coefficient, and β_ij_ is the interaction coefficient. The relation between the coded and the actual can be determined as follows:

5 $$ Xi=\frac{Ui-Uio}{\varDelta Ui} $$

where *Xi* is the coded value of the *i*th variable, *Ui* is the actual value of the *i*th variable, *Ui*0 is the actual value of the *i*th variable at the center point, and Δ*Ui* is the step change of variable.

The data obtained from RSM were subjected to the analysis of variance (ANOVA). The quality of fit of the second-order model equation was expressed by the regression coefficient *R* ^2^ and its statistical significance was determined by an *F*-test. The significance of each regression coefficient was determined using Student’s *t*-test. Three-dimensional plots and their respective contour plots were obtained based on the effects of the levels of two parameters and their interactions on the production of the bioactive compound by keeping the other two parameters at their optimal concentrations. The computer software used for the experimental designs and statistical analysis of the experimental data was Design-Expert version8.0 (Stat-Ease Inc., Minneapolis, U. S. A.).

### Validation of the model

In order to confirm the optimized culture conditions, all experiments were performed in triplicates and results represented the mean values of three independent experiments.

### Characterization of the purified metabolite

The bioactive metabolite was characterized with respect to thermal and pH stability. Susceptibility of the compound to different temperatures was ascertained according to the method described by Lee et al. [52]. pH stability was estimated after 4 h of storage at 4 °C in the following buffers: 50 mM sodium acetate buffer (pH3-5), 50 mM phosphate buffer (pH6-7), Tris–HCl buffer (pH8-9). The samples were then neutralized followed by agar diffusion assay. pH measurement was done with a Eutech pH700 pH meter, calibration was done at the appropriate temperature with standard solutions (pH7.4 and 10) provided with the instrument. Determination of antibiotic activity was done by measurement of the zones of inhibition; measurements were taken from the edge of the antibiotic disk to the margin of the zone [24].

## Abbreviations

CCD, central composite design; PBD, Plackett–Burman design; RSM, response surface methodology
